# Pectoralis Major (Breast) Muscle Extracellular Matrix Fibrillar Collagen Modifications Associated With the Wooden Breast Fibrotic Myopathy in Broilers

**DOI:** 10.3389/fphys.2020.00461

**Published:** 2020-05-07

**Authors:** Sandra G. Velleman

**Affiliations:** Department of Animal Sciences, The Ohio State University, Wooster, OH, United States

**Keywords:** collagen, fibrosis, muscle, necrosis, wooden breast

## Abstract

The poultry industry has placed significant emphasis on the selection of meat-type broilers for increased body weight, increased meat yield especially the pectoralis major (breast) muscle, decreased time to processing, and improved feed conversion_._ Although significant improvements have occurred in fast-growing meat-type broilers, myopathies affecting meat quality especially in the pectoralis major muscle have occurred. Many of the broiler breast muscle myopathies are caused by inflammation leading to the necrosis of existing muscle fibers and resulting in replacement of the muscle fibers with extracellular matrix proteins especially fibrillar collagens, fibrosis. This review explores how the fibrotic deposition and organization of extracellular matrix proteins especially the fibrillar collagens, Types I and III, affects the phenotype of the Wooden Breast myopathy, functional properties of the pectoralis major muscle, and meat quality.

## Introduction

Broilers have been selected for increased growth, carcass weight, pectoralis major (breast) muscle yield, decreased time to reach processing weight, and improved feed conversion (Havenstein et al., 1994a, b, 2003; Collins et al., 2014). Despite the overall growth improvements and increased feed efficiency, the presence of novel necrotic/fibrotic myopathies has been identified. These myopathies negatively impact breast meat quality and affect the well-being and overall function of the pectoralis major muscle. Typical named necrotic/fibrotic myopathies include, but are not limited to, Wooden Breast (WB: Sihvo et al., 2014), White Stripping (WS: Kuttappan et al., 2013), and Spaghetti Meat (SM: Baldi et al., 2018, 2019). All three of these myopathies affect the visual appearance of the pectoralis major muscle with increased collagen deposition, and altered fat and protein levels. Wooden Breast and SM have the most detrimental effects on breast meat quality with WB affected meat being hard whereas SM meat is soft after cooking. In modern heavy weight fast-growing broilers, the pectoralis major muscle is eight times larger than broilers marketed in 1955 (Collins et al., 2014). Selection favoring greater pectoralis major muscle growth is due to consumer preference for a low-fat protein choice that is reasonable in cost. The increase in muscle mass is correlated with larger myofiber diameters from hypertrophic growth (Dransfield and Sosnicki, 1999), and a decrease in connective tissue spacing between muscle fiber bundles (perimysium) and individual myofibers (endomysium) (Yost et al., 2002; Velleman et al., 2003). The presence of ample connective tissue spacing is necessary for structure and function of the muscle and vascularization. Since the pectoralis major is an anaerobic muscle, the removal of lactic acid the by-product of anaerobic respiration requires vascular supply.

The fibrotic process of the replacement of muscle fibers with connective tissue is induced by chronic muscle fiber damage, necrosis, with associated tissue inflammation. In normal muscle, the damage will be repaired without changes in the myofiber structure or fibrosis. However, fibrosis results in muscles with chronic necrosis leading to the excessive deposition of fibrillar collagen as is observed with myopathies like WB. It is not the concentration of fibrillar collagen synthesized during fibrosis, but the organization of the fibrillar collagen will determine the tissue flexibility and the tenderness of the meat. In recent studies, it has been shown that there may be multiple necrotic/fibrotic myopathies associated with the broiler pectoralis major muscle (Velleman, 2015, 2019; Velleman and Clark, 2015; Clark and Velleman, 2017; Velleman et al., 2017). Many of these myopathies, largely go phenotypically undetected by palpation due to differences in the organization of fibrillar collagens. This review focuses on the organization of fibrotic fibrillar collagen deposited in the extracellular connective tissue spaces in the broiler pectoralis major muscle in 3 heavy weight fast-growing commercial broiler with differing incidence of phenotypically detectable WB.

### What Is the Extracellular Matrix and Why It Is a Key Element Determining Muscle Function and Meat Quality

The extracellular matrix is defined as all the secreted molecules extrinsic to the cell composed of collagens, proteoglycans, and non-collagenous glycoproteins. The composition and structure of the extracellular matrix is not random as it is tissue-type and age specific. The matrix is thus dynamically expressed and directly impacts muscle cell proliferation, adhesion, migration, and the repair of damaged muscle fibers. In skeletal muscle there are three layers of connective tissue containing extracellular matrix macromolecules. They are the endomysium, perimysium, and epimysium. The epimysium forms a sheath around the entire muscle, the perimysium encompasses muscle fiber bundles, and the endomysium surrounds individual muscle fibers. The predominant extracellular matrix proteins in these layers are the fibrillar collagens especially Types I and III.

The fibrillar collagens are characterized by a single triple-helical domain containing three peptide chains forming an alpha helix. After the triple helical structure is formed, the collagen is secreted into the extracellular matrix space where it is aligned into a quarter stagger array leading to the formation of collagen fibrils that are stabilized by crosslinking between the collagens. Crosslinking of the fibrillar collagens is necessary for both its structural stability and functional properties and is a major determinant of meat textural properties. The covalent hydroxylsylpyridinoline (HP) crosslink is a mature, non-reducible, trivalent crosslink that forms from the condensation of two divalent ketoimine crosslinks (Reiser et al., 1992). The formation of HP crosslinks is progressive with age and the toughening of meat is directly attributable to crosslink concentration. With tissue injury, the repair process results in collagen fibrils that have higher levels of HP crosslinking (Zimmerman et al., 2001). Since collagen HP crosslinking is progressive with age and increases with tissue injury, fibrotic myopathies will likely result in increased collagen deposition and crosslinking like what is observed in the WB myopathy. Despite the likely increase in collagen crosslinking in severely affected WB muscle, Baldi et al. (2019) showed that hydroxylysylpyridinoline concentration, the principle non-reducible crosslink in fibrillar collagen is not affected in the Ross 308 strain. The muscle necrotic and fibrotic process is characterized by both changes in tissue structure and composition of the extracellular matrix. In fibrotic tissue, there is an excessive deposition of fibrillar collagen (Alexakis et al., 2007; Serrano and Muňoz-Cánoves, 2010). Thus, necrotic and fibrotic conditions in skeletal muscle will result in altered structural architecture and function with reduced elasticity. Furthermore, there is a general replacement of skeletal muscle fibers with connective tissue. Meat derived from pectoralis major muscles with necrotic and fibrotic disorders like WB will have reduced myofibrillar protein content, reduced water holding capacity, increased fat, and be tough and texturally unappealing (Mazzoni et al., 2015).

### Overview of Skeletal Muscle Development and Regeneration

The predisposition to the development of degenerative breast muscle myopathies has its origins in the formation of muscle and its continued growth as well as how selection for fast-growing heavy weights lines has altered the development and growth of the pectoralis major muscle including the extracellular matrix environment. The development and growth of skeletal muscle is a precisely regulated process with specific phases. Embryonic myoblasts are derived from the somites and once they migrate to the areas of muscle formation will further proliferate, align to form multinucleated myotubes, and ultimately muscle fibers. After the myoblasts form multinucleated myotubes they withdraw from the cell cycle. During the formation of skeletal muscle, the muscle fibers will form bundles with the bundles being separated by perimysial connective tissue spacing and the individual muscle fibers by endomysial connective tissue. At the time of hatch, muscle fiber formation is complete (Smith, 1963).

The continued posthatch growth is from the enlargement or hypertrophy of existing muscle fibers. Hypertrophy is solely dependent upon a mesodermally derived stem cells population of adult myoblasts, satellite cells. With myofiber injury, the satellite cells are responsible for the repair and regeneration of the myofiber back to its original state.

Having enough perimysial and endomysial connective tissue spacing is necessary for the livability of the avian pectoralis major muscle myofibers (Wilson et al., 1990). The perimysium is a connective layer composed of groups of collagen fibrils tightly packed that surround the entire muscle fiber bundle. In comparison, the endomysium contains a thin layer of collagen encompassing only individual myofibers. In addition to providing space between the muscle fiber bundles and myofibers, the intramuscular areas of connective tissue spacing provide structural support for the tissue, defines the elasticity or stretch of the muscle, and contains capillaries necessary for adult myoblast activity, satellite cells, and the removal of respiration by-products like lactic acid. Selection for increased breast muscling based on myofiber hypertrophy and not muscle fiber number through hyperplasia will result in myofibers and muscle fiber bundles that occupy the endomysial and perimysial spaces, respectively. As the connective spaces are diminished in size and the fibers and fiber bundles begin to touch, fiber degeneration ensues (Wilson et al., 1990; Velleman et al., 2003). Once the muscle fibers are damaged, satellite cell-mediated repair mechanisms are invoked. When myofiber degeneration occurs the sarcolemma (myofiber plasma membrane) is disrupted which initiates necrosis from the influx of calcium from the sarcoplasmic reticulum. The necrosis of the muscle fibers leads to an immune response (Orimo et al., 1991) with the infiltration of immune cells including neutrophils and macrophages to phagocytize the cellular debris. In necrotic/fibrotic disorders like WB the pectoralis major muscle tissue has observable lysis of existing muscle fibers with immune cell infiltration.

**FIGURE 1:**
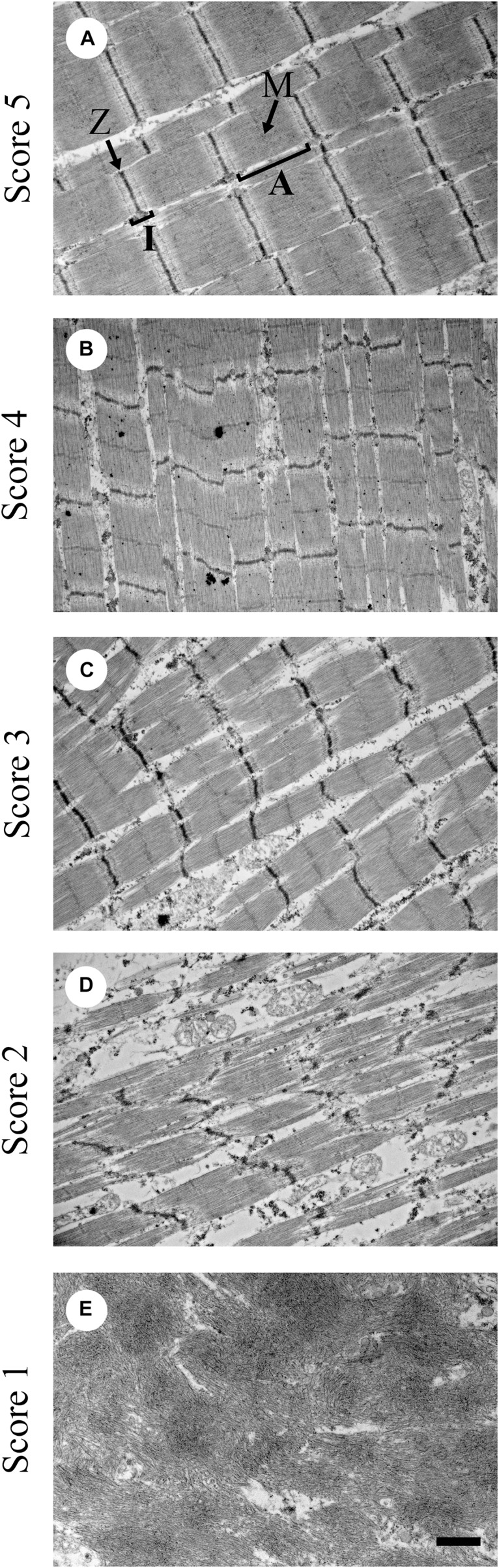
Representative transmission electron microscopy images for sarcomere organization scoring from 1 to 5. **(A)** Score of 5 shows sarcomeres with normal structure; **(B)** score of 4 means mildly altered; **(C)** score of 3 means further disorganized from a score of 4 but still moderate; **(D)** score of 2 means severely altered sarcomere structure; and **(E)** score of 1 means complete absence of sarcomere structure. A = A zone; I = I zone; M = M-line; Z = Z-line. Bar = 1 μm. (Figure reproduced from Velleman et al., 2018. *Avian Dis.* 62, 28–35).

The degeneration or necrosis of the muscle fibers will initiate satellite cell repair mechanisms. Satellite cells are, in general, quiescent and must be activated to reenter the cell cycle to proliferate and differentiate. Satellite cells require the appropriate niche environment for activation. For satellite cell activity to occur, the muscle stem cell niche must contain vascularization within 21 μm of the satellite cells (Christov et al., 2007). However, in meat-type broilers affected with WB the muscle is under oxidative stress (Abasht et al., 2016). One of the characterizing features of WB muscle is a reduction in circulatory supply. The reduction in circulatory supply in the pectoralis major muscle is further augmented by the pectoralis major muscle being an anaerobic muscle. Anaerobic glycolytic metabolism does not require oxygen. Thus, the pectoralis major muscle being a fast twitch Type II muscle does not require by nature an extensive circulatory network. Further reduction in circulatory supply in WB affected muscle will suppress satellite cell-mediated myofiber regeneration. Regeneration is a process of constructing parallel arrays of microfibrils. Alterations in the reconstruction of the myofibrillar structure will negatively impact the contractile properties of the muscle (Velleman et al., 2018) and meat quality by reducing protein content. Velleman et al. (2018) showed that the WB condition resulted in a deposition of smaller diameter myofibrils with a lack of normal sarcomere structure ranging from moderate to severe (Figure 1). In contrast, during normal posthatch muscle growth, myofiber diameter should continue to increase with age through satellite cell-mediated hypertrophic growth (Moss and LeBlond, 1971) while maintaining normal sarcomere structure in the myofibrils.

The degeneration of muscle fibers results in inflammation of the pectoralis major muscle tissue leading to an increased deposition of extracellular matrix proteins like collagen and proteoglycans. The extracellular matrix proteins form an architectural network outside the cell which determines, in part, the structural stability of the tissue, stretch, and water-holding capacity. Measures of extracellular matrix protein concentration alone will not determine the functional attributes of the tissue. For example, the primary phenotypic feature of WB is a hard pectoralis major muscle detected by palpation (Sihvo et al., 2014). As shown by Velleman and Clark (2015), the phenotypic incidence of WB does not align with microscopic assessment for necrosis and fibrosis with the incidence being significantly higher when evaluated microscopically.

### Formation and Organization of Intramuscular Collagen Fibrils: Relationship to the Phenotype of Wooden Breast

The process of fibrosis is a self-perpetuating response to muscle necrosis resulting in the progressive overproduction of fibrillar collagens Types I and III in the perimysial and endomysial connective tissue spaces. The phenotypic characteristics of necrotic/fibrotic myopathies is not a direct relationship to the concentration of these collagens. There are numerous factors determining the tissue phenotype resulting from fibrosis. These include, but are not limited to fibril diameter, degree of crosslinking, proteoglycan localization and type, fibril alignment, and morphometric organization of the collagen fibrils.

All collagens are composed of three polypeptide chains with the amino acid repeat Glycine-X-Y where X and Y are any amino acid but are frequently proline or lysine. The polypeptide chains will wrap around each other intracellularly to form a right-handed triple helix. At this point, the collagen molecule is exocytosed into the extracellular space where fibril and fiber formation take place. The collagen fiber is the functional form impacting tissue structure, elasticity, and ultimately meat quality. After secretion, the collagen molecules will align in parallel to forming a quarter staggered array which is a necessary step leading to the assembly of collagen fibrils. The collagen fibrils are stabilized by the formation of reversible divalent crosslinks. The alignment of the collagen molecules is not a random process and after alignment there are gap and overlap areas within the quarter staggered array. The length of one overlap zone is 67 nm and this is termed a D banding-period. If the alignment of the collagen molecules is altered, the length of the D-period will be modified affecting collagen fibril function. After collagen fibril formation, the collagen fibrils will come together forming collagen fibers. With maturation, divalent ketoimine crosslinks are replaced with trivalent non-reversible HP crosslinks. The HP crosslink is a critical factor in tissue stiffening. After three collagen triple helices are linked together, additional helices are linked together increasing collagen fibril diameter and crosslinking. The formation of HP crosslinks is a progressive process with age and is likely a major factor in the phenotypic detection of WB by palpation and the reduction in meat quality.

**FIGURE 2:**
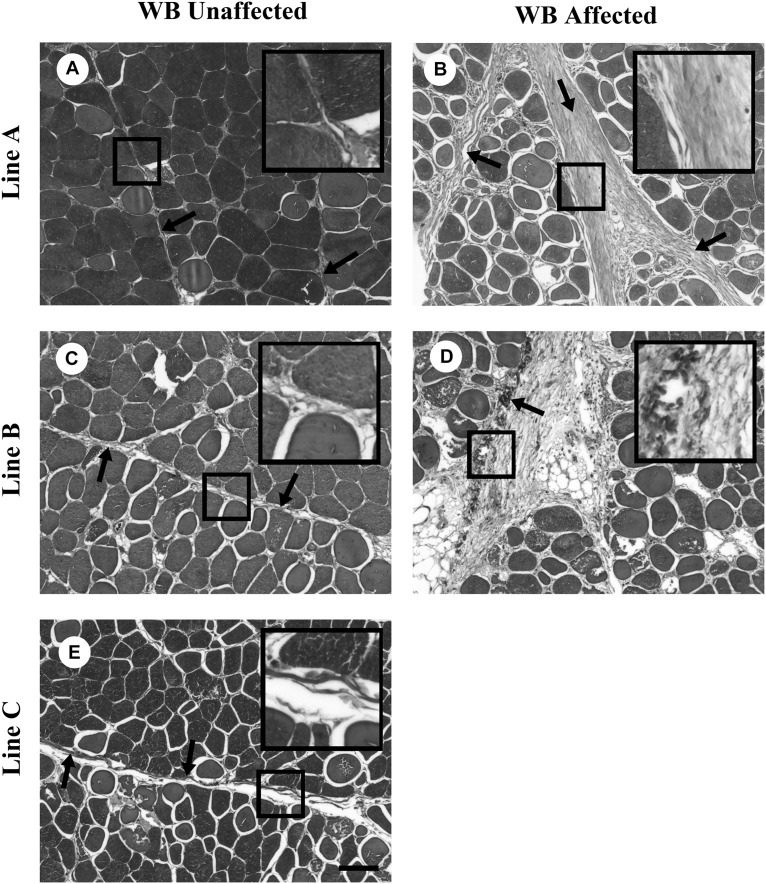
Masson trichrome staining of collagen organization in wooden breast (WB)-unaffected and -affected pectoralis major muscle. **(A,C,E)** are representative images of WB-unaffected pectoralis major muscle from Lines A, B, and C, respectively. **(B,D)** are representative images of WB-affected pectoralis major muscle from Lines A and B, respectively. The arrows highlight fibrillar collagen and the boxes contain enlargements of the fibrillar collagen. Scale bar = 100 μm. (Figure reproduced from Velleman et al., 2017. *Avian Dis.* 61, 481–490).

Since the WB myopathy is characterized by excessive collagen fibril deposition, it is of importance to understand the ultrastructural intramuscular organization of the fibrillar collagens in fast growing meat-type broiler lines of differing parental lineage. To comprehensively study collagen fibril structure, a series of studies was conducted examining collagen fibril structure in three fast growing commercial broiler lines with distinctly different levels of phenotypically detectable WB (Velleman and Clark, 2015; Velleman et al., 2017; Tonniges et al., 2019). These lines will be referred to as A, B, and C. Line C does not exhibit any WB whereas Line A has a high degree of affected birds, and Line B has an intermediate level of phenotypic detection of WB affected birds. Velleman and Clark (2015) and Velleman et al. (2017) using light microscopy observed that Line A with a high degree of phenotypically WB affected birds, had perimysial collagen fibers characterized by parallel packing (Figure 2). In contrast, Line B by palpation had a low percentage of birds categorized as WB affected. Light microscopic examination revealed that the perimysial collagen was not packed but diffuse in structure. Furthermore, histological examination of the pectoralis major muscle showed that 70% of the Line B birds had microscopically observable necrosis and fibrosis. Therefore, phenotypic palpation for breast muscle hardness, a standard approach used by the broiler industry, is an insufficient measure for pectoralis major muscle fibrosis.

Transmission electron microscopy (TEM) analysis was used to further investigate histological differences, measure collagen D-periodicity, and collagen fiber diameter in Lines A, B, and C affected and unaffected WB birds (Velleman et al., 2017). Velleman et al. (2017) showed that WB affected Line A muscle had overall smaller average collagen fibril diameter and longer average collagen D-period compared to unaffected Line A muscle. Line B showed no such differences. Fibrotic collagen of WB-affected muscle of Line A exhibited D-period length and fibril diameter more like endomysial collagen of unaffected muscle of Line A. However, the fibrillar structure of fibrotic collagen in Line B affected with WB was similar to perimysial collagen of Line B-unaffected muscle. The endomysial collagen of Line A was changed with the WB myopathy while the endomysial collagen of Line B was not. Endomysial collagen of Line A exhibited decreased fibril diameter and increased D-period length with WB. Changes in D-period length and collagen fibril diameter are a direct consequence of the arrangement of collagen within the collagen fibril. Modifications of the collagen fibril are suggestive of changes in the molecular packing of collagen monomers, which will affect collagen function by altering protein binding sites or the flexibility of the collagen fibril.

More importantly the TEM study by Velleman et al. (2017) demonstrated that there are different fibrotic collagen organizations in the pectoralis major muscle of fast-growing meat type broilers. These differences in fibrosis are likely unique myopathies. Myopathies like WB have increased stiffness or hardness of the pectoralis major muscle likely due to high levels of collagen crosslinking which results in tightly packed collagen fibrils. Fibrotic myopathies undetected by palpation is due to the collagen fibrils being diffuse with lower levels of crosslinking, and the pectoralis major muscle remains texturally soft and in extreme case SM may be found. Despite not being phenotypically detectable, necrosis and fibrosis will still alter the nutritional value of the breast meat by reducing protein levels and increasing fat concentration.

Since the organization of collagen fibrils is altered by the fibrotic process, it is necessary to determine mechanistically how this organizational change occurs. A primary candidate for altering collagen fibrillar structure is the chondroitin and dermatan sulfate proteoglycan decorin. Decorin plays a critical role in the formation of the quarter stagger array aligning the collagen helices (Weber et al., 1996) and subsequent HP crosslinking (Reiser et al., 1992) by binding to the D banding period. Extracellular matrix organization has been implicated in other fibrotic conditions including but not limited to lung fibrosis (Blaauboer et al., 2014) and human renal fibrosis disease (Stokes et al., 2000). Velleman and Clark (2015) reported a high correlation of decorin expression in WB affected muscle in Line A but not in Line B.

**FIGURE 3:**
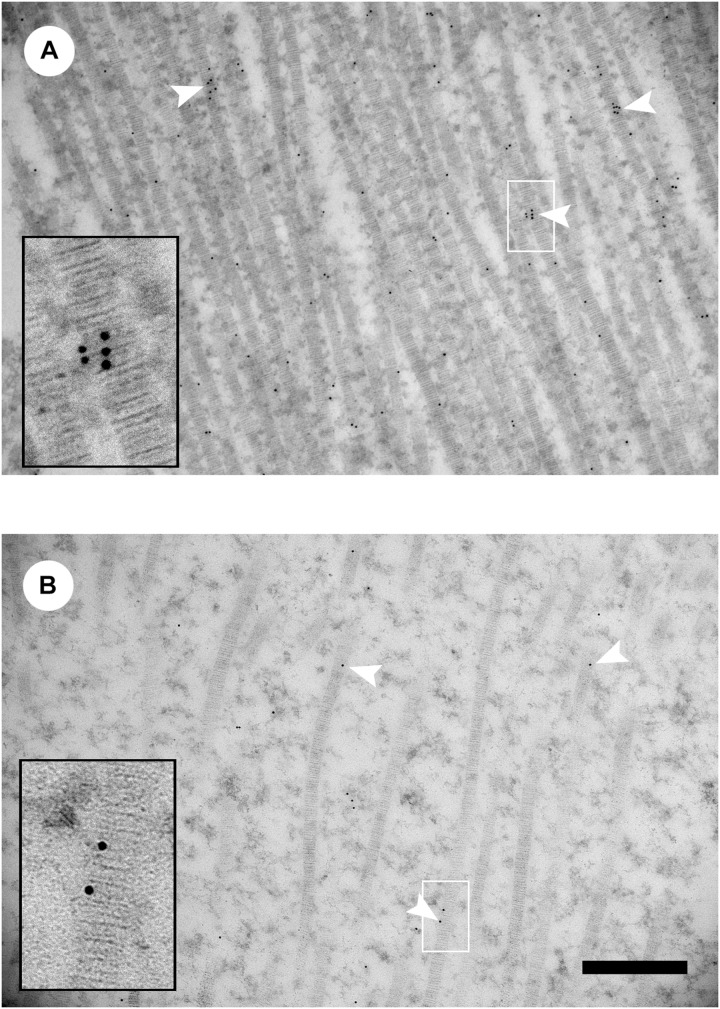
Decorin immunogold transmission electron microscopy images of collagen fibrils from wooden breast-affected muscle of Line A **(A)** and Line B **(B)**. White arrowheads indicate gold particles labeling collagen fibrils. Insets show enlarged images of gold labeling individual collagen fibrils in the region indicated by the white box. Scale bar = 500 nm. (Figure reproduced from Tonniges et al. (2019). *Avian Dis.* 63, 48–60).

Tonniges et al. (2019) investigated the association of decorin binding and collagen organization in Lines A, B, and C using TEM and immunogold labeling decorin. The WB myopathy caused larger diameter collagen fibril bundles. However, Line A with the high phenotypic detection of WB had larger collagen fibril bundles compared to WB affected pectoralis major muscles in Line B. Line A had collagen fibril bundles upto 8.4 μm and Line B had a maximum diameter of 5.1 μm. It was further found that Line A WB affected muscle had smaller diameter collagen fibril bundles with more decorin collagen binding than Line B. Figure 3 shows a representative TEM decorin immunogold image for Lines A and B. In addition to the observable differences in decorin-collagen binding, the images also illustrate the distinct differences in collagen organization.

To summarize this review provides novel insight into the fibrillar organization of collagen in the broiler pectoralis major muscle and its impact on the phenotypic detection of WB affected birds. Despite Tonniges et al. (2019) reporting that smaller diameter collagen fibril bundles in WB affected muscle having more decorin-collagen binding, it is unlikely for decorin to play a major role in the alignment and organization fibrotic fibrillar WB collagen. In contrast decorin knockout studies in mice demonstrated irregular collagen fiber organization was directly associated with fragility of tissues like the skin tearing with force (Danielson et al., 1997). The presence of multiple fibrotic myopathies in the broiler pectoralis major muscle is likely associated with the expression and organization of Types I and III collagen.

Skeletal muscle contains fibrillar collagens Types I and III. The expression of collagen is age dependent. During early development, Type III collagen is the predominant collagen but with increased age there is a shift toward Type I collagen (Bornstein and Sage, 1980; Light and Champion, 1984; Kovanen and Suominen, 1989). With injury and inflammation as occurs in myopathies like WB, the expression of collagen will shift back to more of an embryonic-like tissue with increased levels of Type III collagen. As the tissue is repaired Type I collagen expression will then increase and Type III collagen will decrease (Bailey et al., 1975; Barnes et al., 1976; Weber et al., 1978; Merkel et al., 1988). Collagen fibrils can be homotypic composed of only a single collagen type or heterotypic containing a mixture of collagen types. Although the functional impact of collagen fibril composition is not well understood, it has been postulated that collagen fibril composition can influence muscle stiffness and meat toughness (McCormick, 1994). Studies investigating differential collagen fibril composition and its impacts has not been reported to date for broiler skeletal muscle. In other tissue systems, heterotypic collagen fibrils containing both Types I and III collagen have reduced stiffness and collagen fibril diameter (Romanic et al., 1991; Notbohm et al., 1993; Asgari et al., 2017). Interestingly, when collagen Type III expression is increased as observed with skeletal muscle injury (Hurme et al., 1991; Gibertini et al., 2014), Type III collagen is localized in highly aligned and tightly packed collagen fibrils (Brisson et al., 2015; McConnell et al., 2016) as observed in the phenotypically detectable WB. Thus, collagen Type III expression may be associated with the fibrotic organization of collagen fibrils. Future studies will need to address collagen fibril composition and its association with broiler pectoralis major muscle fibrosis.

## Author Contributions

The author confirms being the sole contributor of this work and has approved it for publication.

## Conflict of Interest

The author declares that the research was conducted in the absence of any commercial or financial relationships that could be construed as a potential conflict of interest.
